# Kinetics of Nucleocapsid, Spike and Neutralizing Antibodies, and Viral Load in Patients with Severe COVID-19 Treated with Convalescent Plasma

**DOI:** 10.3390/v13091844

**Published:** 2021-09-15

**Authors:** Thomas P. Thomopoulos, Margherita Rosati, Evangelos Terpos, Dimitris Stellas, Xintao Hu, Sevasti Karaliota, Anthi Bouchla, Ioannis Katagas, Anastasia Antoniadou, Andreas Mentis, Sotirios G. Papageorgiou, Marianna Politou, Jenifer Bear, Duncan Donohue, Anastasia Kotanidou, Ioannis Kalomenidis, Eleni Korompoki, Robert Burns, Maria Pagoni, Elisavet Grouzi, Stavroula Labropoulou, Kostantinos Stamoulis, Aristotelis Bamias, Sotirios Tsiodras, Meletios-Athanasios Dimopoulos, George N. Pavlakis, Vasiliki Pappa, Barbara K. Felber

**Affiliations:** 1 Hematology Unit, Second Propaedeutic Department of Internal Medicine and Research Institute, School of Medicine National and Kapodistrian University of Athens, University General Hospital “Attikon”, 18120 Athens, Greece; ththomop@med.uoa.gr (T.P.T.); anthibouhla@hotmail.com (A.B.); ioanniskatagas@gmail.com (I.K.); sotirispapageorgiou@hotmail.com (S.G.P.); abamias@med.uoa.gr (A.B.); 2 Human Retrovirus Section, Vaccine Branch, Center for Cancer Research, National Cancer Institute, Frederick, MD 21702, USA; margherita.rosati@nih.gov (M.R.); dimitrios.stellas@nih.gov (D.S.); sevasti.karaliota@nih.gov (S.K.); george.pavlakis@nih.gov (G.N.P.); 3 Department of Clinical Therapeutics, School of Medicine, National and Kapodistrian University of Athens, 11528 Athens, Greece; eterpos@med.uoa.gr (E.T.); e.korompoki@imperial.ac.uk (E.K.); mdimop@med.uoa.gr (M.-A.D.); 4 Human Retrovirus Pathogenesis Section, Vaccine Branch, Center for Cancer Research, National Cancer Institute, Frederick, MD 21702, USA; xth38302@gmail.com (X.H.); jenifer.bear@nih.gov (J.B.); robert.burns@nih.gov (R.B.); barbara.felber@nih.gov (B.Κ.F.); 5 Frederick National Laboratory for Cancer Research, Basic Science Program, Frederick, MD 21701, USA; 6 Fourth Department of Internal Medicine, School of Medicine, University General Hospital “Attikon”, National and Kapodistrian University of Athens, 11527 Athens, Greece; ananto@med.uoa.gr (A.A.); tsiodras@med.uoa.gr (S.T.); 7 National Influenza Reference Laboratory of Southern Greece, Hellenic Pasteur Institute, 11521 Athens, Greece; mentis@pasteur.gr (A.M.); vlabropoulou@pasteur.gr (S.L.); 8 Hematology Laboratory-Blood Bank, Aretaieion Hospital, School of Medicine, National and Kapodistrian University of Athens, 11528 Athens, Greece; mariannapolitou@gmail.com; 9 MS Applied Information and Management Sciences, Frederick National Laboratory for Cancer Research, Frederick, MD 21702-1201, USA; duncan.donohue@nih.gov; 10 First Department of Critical Care Medicine and Pulmonary Services, Evangelismos General Hospital, National and Kapodistrian University of Athens, 11527 Athens, Greece; akotanid@gmail.com (A.K.); ikalom@med.uoa.gr (I.K.); 11 BMT Unit, Haematology-Lymphomas Department, Evangelismos Hospital, 10676 Athens, Greece; marianpagoni@yahoo.com; 12 Transfusion Service and Clinical Hemostasis of Saint Savvas, Oncology Hospital of Athens, 11522 Athens, Greece; egrouzi@otenet.gr; 13 Hellenic National Blood Transfusion Center, 13678 Athens, Greece; kostas.stamoulis@gmail.com

**Keywords:** convalescent plasma, SARS-CoV-2, COVID-19, antibody kinetics, neutralizing antibodies, spike, nucleocapsid

## Abstract

COVID-19 is an ongoing pandemic with high morbidity and mortality. Despite meticulous research, only dexamethasone has shown consistent mortality reduction. Convalescent plasma (CP) infusion might also develop into a safe and effective treatment modality on the basis of recent studies and meta-analyses; however, little is known regarding the kinetics of antibodies in CP recipients. To evaluate the kinetics, we followed 31 CP recipients longitudinally enrolled at a median of 3 days post symptom onset for changes in binding and neutralizing antibody titers and viral loads. Antibodies against the complete trimeric Spike protein and the receptor-binding domain (Spike-RBD), as well as against the complete Nucleocapsid protein and the RNA binding domain (N-RBD) were determined at baseline and weekly following CP infusion. Neutralizing antibody (pseudotype NAb) titers were determined at the same time points. Viral loads were determined semi-quantitatively by SARS-CoV-2 PCR. Patients with low humoral responses at entry showed a robust increase of antibodies to all SARS-CoV-2 proteins and Nab, reaching peak levels within 2 weeks. The rapid increase in binding and neutralizing antibodies was paralleled by a concomitant clearance of the virus within the same timeframe. Patients with high humoral responses at entry demonstrated low or no further increases; however, virus clearance followed the same trajectory as in patients with low antibody response at baseline. Together, the sequential immunological and virological analysis of this well-defined cohort of patients early in infection shows the presence of high levels of binding and neutralizing antibodies and potent clearance of the virus.

## 1. Introduction

Coronavirus disease 19 (COVID-19) is an infectious disease caused by the newly emerged Severe Acute Respiratory Syndrome Coronavirus 2 (SARS-CoV-2). Since its discovery in Wuhan, China in December 2019, it has caused an ongoing pandemic [1]. Among the structural proteins of SARS-CoV-2, Spike (S) and Nucleocapsid (N) have been recognized as the most immunogenic [2].

Regarding the natural history of COVID-19, after an incubation period of up to 14 days following transmission, most patients develop generally mild non-specific symptoms of upper respiratory tract infection, including anosmia, ageusia, and rhinitis, as well as lower respiratory tract infection symptoms, including dyspnea and cough; however, in some patients, this stage is followed by rapid deterioration, usually within 7–10 days of symptom onset, characterized by a hyperinflammatory syndrome, acute respiratory distress syndrome (ARDS), and end-organ damage; hypercoagulability is also prevalent at this stage, and patients might experience a high risk of pulmonary embolism [3].

Despite rigorous research, therapeutic options for COVID-19 remain limited, as only the administration of dexamethasone has demonstrated a survival benefit both in randomized control trials and observational studies [4], whereas the efficacy of remdesivir or other antiviral agents remains limited [5]. Most recently, the US Food and Drug Administration (FDA) granted Emergency Use Authorization for two neutralizing antibody cocktails (casirivimab/imdevimab and bamlanivimab/etesevimab), as well as monotherapy with bamlanivimab for treatment of ambulatory patients who have a high risk of progressing to severe disease [6]. On the other hand, individual randomized control trials on the efficacy of convalescent plasma (CP) have shown negative results [7,8,9,10,11,12,13,14,15,16,17]; however, two recent meta-analyses have demonstrated the efficacy of convalescent plasma (CP) in mortality reduction of patients with severe COVID-19, similarly to the results of several matched-control trials [18,19]. A survival benefit of patients treated with CP was recently shown by our group in a matched propensity score analysis of 59 patients [20].

Despite the increasing understanding of the humoral response in COVID-19 [21], very little is known regarding the antibody response in patients who have been treated with CP; therefore, herein, we opted to explore the antibody kinetics of patients with severe COVID-19 who received CP within a phase II multicenter trial in Greece.

## 2. Materials and Methods

### 2.1. Study Design

This study was part of a multicenter prospective phase II trial (identifier number NCT04408209), conducted at five hospitals in Athens, Greece. All study procedures were carried out in accordance with the declaration of Helsinki; the study was also approved by the local ethics committees of all participating hospitals. All patients provided written informed consent. Details regarding the study protocol, including the inclusion and exclusion criteria, have been described elsewhere [20]. In short, following informed consent, patients with severe COVID-19 received single-donor CP, divided into three equal doses and infused on days 1, 3, and 5. Clinical and laboratory parameters were recorded daily for the first seven days and on a weekly basis until day 28.

An in-house ELISA measuring IgG was used to determine antibodies against the complete Spike protein, Spike-Receptor binding domain (Spike-RBD), complete Nucleocapsid protein (N), and Nucleocapsid-RNA binding domain (N-RBD), as previously described. The in-house assay measuring Spike shows excellent correlation with the ROCHE assay but has a larger range of detection and is more sensitive [22,23]. The cut-off values were determined using 17–23 healthy human plasma samples collected between 2015 and 2018 and tested against the different antigens, and the mean and standard deviation were calculated as previously described [22]. A model fit approach was conducted in R, a framework for statistical modeling, to model the curve to define endpoint titers [24]. Neutralizing antibodies (NAb) were determined using a Wuhan-Hu-1 Spike pseudotyped pHIV_NL_ΔEnv-Nanoluc assay, and a 50% inhibitory dose was determined (ID50) [24,25]

A real-time one-step reverse transcription PCR (RT-PCR), specific for the ORF1ab gene of SARS-CoV-2 and the N gene of all other coronaviruses, was performed using the VIASURE SARS-CoV-2 Real Time PCR Detection Kit (CerTest Biotec SL, Zaragoza, Spain) as detailed elsewhere [20]. Briefly, nasopharyngeal swabs collected on days 1, 4, 7, 10, 14, 21, and 28 were analyzed. The Ct values reflecting the number of cycles needed for the first detection of the viral RNA during the real-time PCR reaction were used as an indirect indication of the viral load (higher Ct values reflected lower viral load).

### 2.2. Statistical Analysis

For the determination of the endpoint antibody titers, the right side of the sigmoid dilution curve (all points after the largest drop in measured value or the highest four dilution points, whichever was longer) was fit to a self-starting asymptotic regression model used to determine the nonlinear least-squares estimate of the model parameters, as previously described [22]. Endpoint titers were log10-transformed. Antibody titers and PCR Ct values at different time points were summarized using median and interquartile ranges (IQRs), assuming deviation from normality. A non-parametric Kruskal–Wallis test was used to evaluate the differences of antibody titers from baseline. The correlation between Spike/Spike-RBD and Nabs at different time points was assessed using Spearman’s correlation. All statistical analyses were performed using SPSS version 23.

## 3. Results

### 3.1. Baseline Antibody Levels

From 7 May 2020 to 10 November 2020, 60 patients with WHO grade ≥4 COVID-19 disease were enrolled and received CP transfusion, and the clinical characteristics and outcomes of these patients have been previously described [20]. Among them, 31 patients had at least three consecutive ELISA antibody measurements and viral load data and were included in the current analysis. No statistically significant differences pertaining to age, gender, comorbidities, percentage of infiltrates at baselines, time to study enrollment, baseline antibody titers, or SARS-CoV2 PCR Ct values between the initial and final study populations were documented, demonstrating that no selection bias occurred (data not shown). Baseline clinical parameters, median levels of Spike, Spike-RBD, Nucleocapsid, and N-RBD antibodies, as well as neutralizing antibodies and PCR Ct values, are summarized in Table 1. Eighteen patients had low neutralizing antibodies at baseline (ID50 <1 log), whereas 13 patients had high NAb (ID50 > 1 log).

### 3.2. Kinetics of Binding Antibodies Following CP Infusion

**Figure 1:**
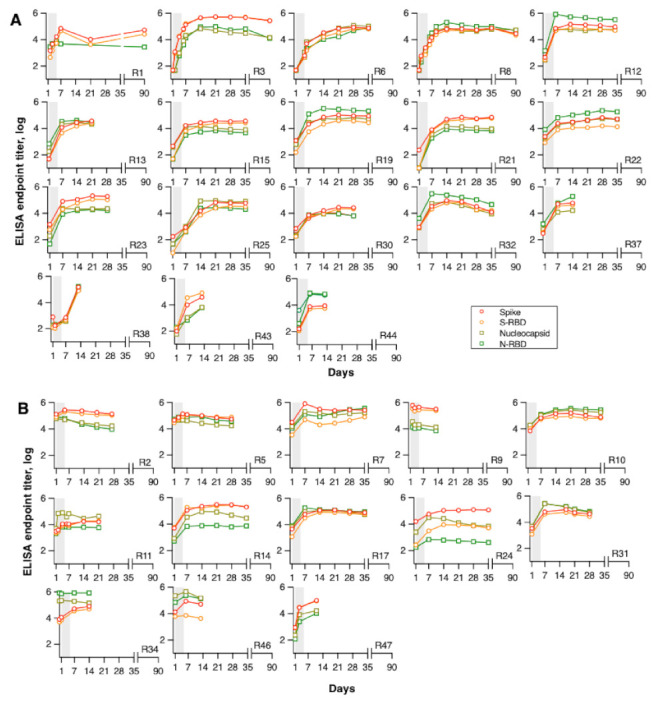
Kinetics of SARS-CoV-2 antibody development in COVID-19 patients treated with convalescent plasma. Antibodies against Nucleocapsid, N-RBD, Spike, and Spike-RBD are plotted as endpoint titers for *n* = 31 patients at indicated timepoints. Patients were separated into two groups on the basis of antibody titers at day 1. (**A**) Patients (*n* = 18) with low Spike (2.5 log) and spike-RBD (2 log) titers and (**B**) patients (*n* = 13) with high Spike (4 log) and Spike-RBD (3.5 log). Grey shaded area marks the time of the 3 convalescent plasma transfusions.

The longitudinal changes in humoral immune responses are shown separately for patients with low antibody titers (Spike median 2.5 log; Spike-RBD median 2 log) (Figure 1A) and high antibody titers (*n*= 13; Spike median 4 log; Spike-RBD median 3.5 log) (Figure 1B) at baseline.

Sequential analysis showed a rapid increase in antibodies in patients with low baseline levels (Figure 1A), reaching peak levels ~7–14 days later. The increase in Spike antibodies paralleled those of Nucleocapsid antibodies. Spike-RBD and N-RBD antibody changes showed the same kinetics as those of Spike and Nucleocapsid. Approximately half of the patients showed higher Spike responses (R3, R15, R21, R23, R43), while others showed higher Nucleocapsid responses (R12, R19, R22, R32, R37, R44), and this hierarchy was maintained at all subsequent time points. Notably, R32 and R37 required mechanical ventilation, were admitted to ICU, and succumbed to the infection.

Patients with high antibodies at baseline also demonstrated subsequent increases, albeit to a lower extent. Several patients had higher Spike antibodies (R2, R9, R14, R47) while others showed higher Nucleocapsid responses (R34, R46) sustained among the studied time points. Both patients with high Nucleocapsid responses at baseline had very severe COVID-19, requiring mechanical ventilation and admission to the ICU; however, they survived to convalescence. Altogether, SARS-CoV-2-infected persons demonstrated distinct patterns of immune responses with a focus on stronger Spike or Nucleocapsid antibodies but maintaining the response hierarchy in the follow-up.

### 3.3. Correlation of Binding Antibodies and Neutralizing Antibodies Following CP Infusion

**Figure 2:**
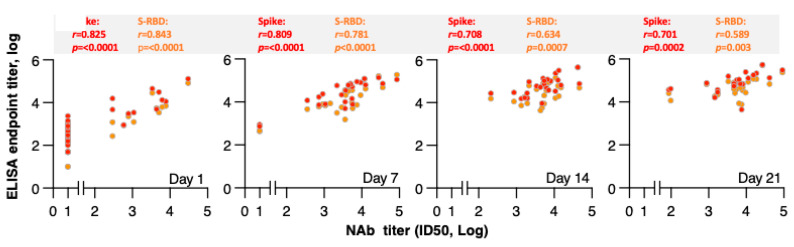
Correlations of the Spike and Spike-RBD binding and Neutralizing antibodies (NAb) at different time points. Correlations of reciprocal pseudotype NAb titers (ID50, log) and reciprocal endpoint titers of Spike (red symbols) and Spike-RBD (orange symbols) measured at indicated time points. Spearman *r* and *p* values are given.

Neutralizing antibodies were monitored over time. Analysis of measurements at days 1, 7, 14, and 21 showed increases in NAb over time, with strong correlations between NAb and Spike and Spike-RBD antibody endpoint titers, respectively, as depicted in Figure 2.

### 3.4. Kinetics of Neutralizing Antibodies and Viral Load Following CP Infusion

**Figure 3:**
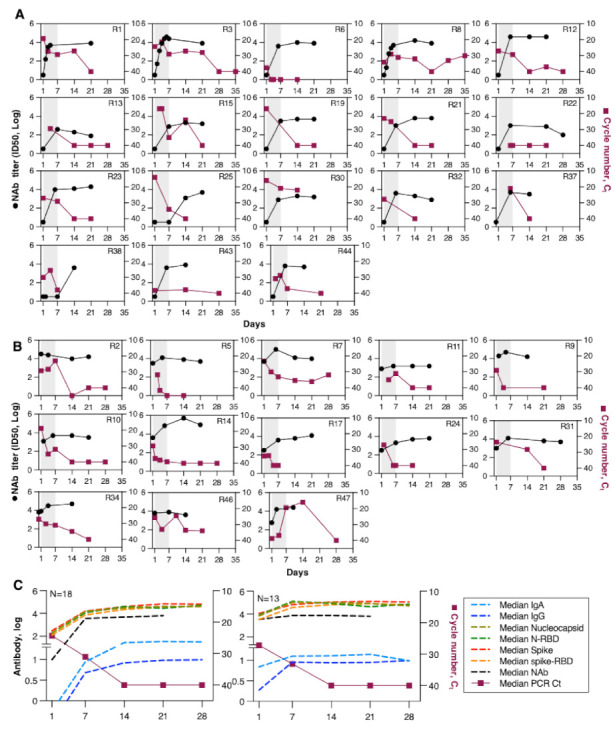
Kinetics of Neutralizing antibody (NAb) and viral load (Ct). NAb and viral loads (Ct values) of patients (*n* = 31) are shown separated as in Figure 1. (**A**) Patients (*n* = 18) with undetectable pseudotype NAb antibodies. (**B**) Patients (*n* = 13) with a median reciprocal pseudotype NAb titer (ID50) of 3.5 log at day 1. (**C**) Median values of reciprocal endpoint titers are shown for Nucleocapsid, N-RBD, Spike, Spike-RBD (from Figure 1), and pseudotype NAb (from panels (**A**,**B**)) indicated on the left axis, and the Ct values are indicated on the right axis. Grey shaded area marks the time of the 3 convalescent plasma transfusions. The median Spike-specific IgA and IgG reported previously [20] are also superimposed, showing similar kinetics.

In addition to binding and neutralizing antibodies, viral loads (Ct value) were measured by RT-PCR from nasopharyngeal swabs over time. Figure 3 shows plots of NAb responses and viral loads of individual patients. The subset of patients that had lower Spike antibodies at day 1 had non-detectable NAb (Figure 3A) and showed high viral loads (median 25 Ct). NAb levels showed rapid increases (Figure 2 and Figure 3A) in parallel with a decrease in viral load to the level of detection (40 Ct) by ~day 14. Patients with detectable NAb had non-significantly lower viral load at enrollment (median 27 Ct) demonstrated, as expected, a smaller increase and slightly faster virus clearance by day 7 (median 33 versus 30 Ct) and virus clearance by day 14 (Figure 3B). Some patients in this group had a much slower viral load decline (R31, R34, R46, R47), despite high levels of antibody responses, which might be attributed to antibody responses of lower function in terms of affinity and neutralizing capacity.

The comparison of changes in binding antibodies recognizing Spike and Nucleocapsid between the two patient subsets (Figure 3C) shows that patients that had higher Ab at day 1 developed significantly higher responses [day 1 and day 7, *p* <0.0001; day 14 0.0015 (Spike), *p* = 0.0008 (Nucleocapsid)] measured by the ELISA assay. Despite significantly higher Spike, Spike-RBD, and NAb in the subset of patients that had higher responses at day 1, both groups showed similar clearance of the virus.

## 4. Discussion

In this study, we evaluated anti-SARS-CoV-2 antibody and viral load kinetics in a well-studied subcohort of 31 patients with severe COVID-19 who were treated with convalescent plasma. This cohort of patients provided us with a unique opportunity to collect several closely spaced samples that allowed us to monitor antibody development in a great number of patients in the very early phase of infection. We showed that patients with undetectable neutralizing antibodies at baseline showed a sharp increase in all anti-SARS-CoV-2-binding antibodies following CP infusion, reaching peak levels within two weeks post-CP. Antibodies recognizing the complete epitopes of trimeric Spike and Nucleocapsid increased in parallel, in great accordance with the increase in antibodies recognizing Spike-RBD and N-RBD, respectively. Neutralizing antibodies were correlated with anti-Spike and anti-Spike-RBD at all time points, and their increase paralleled the viral load clearance, as documented by the concurrent increase in SARS-CoV-2 PCR Ct values. Regarding patients with high immune responses at baseline, a minimal further increase in binding and neutralizing antibodies was documented; however, the viral load showed the same trajectory with clearance within two weeks post-CP.

Notably, patients who developed a more robust immune response against Nucleocapsid compared to Spike had more severe disease and sustained the same pattern throughout the studied period.

To our knowledge, this is the largest study comprehensively evaluating the kinetics of binding antibodies, neutralizing antibodies, and viral load at consecutive time points at the acute phase of COVID-19. Our findings are in line with previous reports on antibody kinetics in CP recipients [26,27,28,29]. Madariaga et al. evaluated anti-RBD and anti-Spike antibodies in ten CP recipients up to 14 days following infusion and demonstrated a 31% and 40.3% increase per day, respectively [26]. Employing similar statistical methods, we demonstrated a comparable increase in a larger sample size with longer follow-up. Duan et al. showed a steep increase in neutralizing antibodies correlating with the disappearance of viral load following CP infusion, also in line with our findings [28]. Conversely, Li et al. demonstrated an increase in Spike and RBD antibodies but not in Nucleocapsid antibodies in 10 patients who received CP [30], in conflict with both our findings and those of a recent study by Arrieta et al., which showed a significant increase in Nucleocapsid antibodies following CP infusion in 10 children with moderate COVID-19 [31]. The strong anti-Nucleocapsid immune response observed in several patients in our study is also in line with recent literature. Tan et al., studying 12 patients longitudinally, demonstrated that early anti-Nucleocapsid response was preferentially induced in patients with severe COVID-19 [32]. Our cohort was comprised exclusively of patients with severe COVID-19, justifying the high prevalence of patients with robust anti-Nucleocapsid response among them. Notably, in our study, two of the three patients who succumbed to COVID-19 had early strong anti-Nucleocapsid immune responses.

Regarding the comparison of immune response between CP recipients and non-CP-treated patients, Klein et al. showed similar Spike-RBD kinetics between a cohort of 34 CP recipients compared to a matched control group; however, in this study, CP was given at a median of 11 days from symptom onset. Delayed intervention might explain the null effect on CP kinetics [33]. Similarly, the PlasmAr study showed increased antibody levels only on day 2 following CP infusion compared to controls, whereas no differences were observed at the subsequent time points evaluated [9]. Therefore, no definite conclusion regarding the contribution of CP to the antibody increase observed in the CP recipients can be easily drawn [34]. It should be noted, however, that in our study, virtually all patients were seropositive by day 7 from the first CP infusion, irrespective of the time after symptom onset or disease severity. This observation contradicts previous research on non-CP-treated patients, which showed that seroconversion naturally occurs 12–13 days post-symptom onset [35]. Moreover, several studies have shown that antibodies against Spike and Nucleocapsid proteins might follow different kinetics, influenced by disease severity [36,37,38,39,40]. The rate of anti-SARS-CoV-2 antibody positivity early in the disease course might be influenced by the severity of COVID-19, as patients with more severe disease tend to have a longer period of seroconversion [39,40]; moreover, the variable extent of early antibody detection early might depend on the methods used and the less well-defined day of symptom onset in different cohorts [41]. Most intriguingly, in our study, Spike and Nucleocapsid antibodies showed a parallel pronounced increase, particularly during the first 7 days from CP infusion. Regarding neutralizing antibodies, an earlier-than-expected peak at seven days post-CP has been noted, also supporting the role of CP in the humoral response [42]. These observations support the contribution of early CP administration to robust humoral responses.

Among the strengths of our study are the large number of participants treated with single-donor CP with known antibody levels, along with the meticulous determination of anti-Spike, anti-Nucleocapsid, and neutralizing antibodies and viral load, at various time points up to day 28, allowing us to compare their kinetics; however, the lack of respective measurements in a matched control group is a limitation of our study, as it prevents us from concluding whether the observed increase in antibodies can be attributed to the CP infusion or whether they represent the normal host immune response to COVID-19. Other limitations of our study include the lack of concurrent assessment of cellular components of the innate and adaptive immunity that might contribute to the early immune response against SARS-CoV-2. Moreover, our in-house ELISA was specific for IgG antibodies; therefore, IgM responses were not evaluated. Most importantly, the study enrollment and subsequent determination of the baseline antibody levels were performed at a median of six days from symptoms onset, and thus our assessment could not capture very early antibody responses to SARS-CoV-2 infection.

## 5. Conclusions

In conclusion, we presented a comprehensive characterization of the kinetics of Nucleocapsid and Spike antibodies in the acute phase in patients who were treated with CP. Spike antibodies increased steeply in parallel with Nucleocapsid antibodies, particularly in patients with no immune response at baseline. Neutralizing antibodies increased in parallel with anti-Spike and anti-Spike-RBD binding antibodies, particularly against Spike and Spike-RBD. Viral load clearance was correlated with a neutralizing antibody increase and occurred within 14 days of symptom onset, irrespective of immune response status at baseline. Finally, the strong anti-Nucleocapsid response might be associated with more severe disease, a finding that merits further research, as it might represent a potential prognostic marker.

## Figures and Tables

**Table 1:** Baseline characteristics, antibody titers, and PCR Ct values in 31 recipients CP recipients.

	CP Recipients (*n*: 31)
Age, median (IQR)	62 (19)
Gender, %	
Female	35.5
Male	64.5
Comorbidities, %	64.5
Percentage of infiltrates at baseline CT, %	
<25	26.7
25–50	46.7
50–75	20.0
≥75%	6.7
Baseline Sequential Organ Failure Assessment (SOFA) score, median (IQR)	5 (3)
Oxygen support, % On air Nasal cannul AVenturi mask Mechanical ventilation	15.0 43.3 31.7 10.0
Time from first symptom to diagnosis, median (IQR) (days)	3 (5)
Time from first symptom to CP infusion, median (IQR) (days)	6 (4)
Time from diagnosis to CP infusion, median (IQR) (days)	3 (3)
Baseline antibody titers, median (IQR)	
Nucleocapsid ^a^	2.7 (1.45)
N-RBD ^a^	2.83 (2.21)
Spike ^a^	2.94 (1.41)
Spike-RBD ^a^	2.53 (1.49)
Neutralizing antibodies ^b^	1.0 (2.01)
PCR Ct value, median (IQR)	25.52 (9.5)

^a^ Endpoint titer, log10; ^b^ ID50, log10.

## Data Availability

The data presented in this study are available on request from the corresponding author.
